# Influence of marital status on the treatment and survival of middle-aged and elderly patients with primary bone cancer

**DOI:** 10.3389/fmed.2022.1001522

**Published:** 2022-10-18

**Authors:** Yixin Wen, Hui Zhang, Kaining Zhi, Minghui Li

**Affiliations:** ^1^Department of Orthopaedics, Fifth Hospital in Wuhan, Wuhan, China; ^2^Department of Blood Transfusion, Wuhan Hankou Hospital, Wuhan, China

**Keywords:** bone cancer, early death, marital status, prognosis, treatment, middle-aged and elderly

## Abstract

**Objective:**

The role of spousal support has been recognized to benefit patients with many chronic diseases and cancers. However, the impact of marital status on the survival of middle-aged and elderly patients with primary bone tumors remains elusive.

**Materials and methods:**

The data of patients aged ≥ 45 years with primary bone tumors diagnosed between 2000 and 2018 were extracted from the Surveillance, Epidemiology, and End Results Database. Kaplan–Meier analysis was used to assess the overall survival and tumor-specific survival of patients. The Cox proportional hazards and Fine-and-Gray models were used to calculate the hazard ratios (HRs) and sub-distribution HRs (sHR) and the corresponding 95% confidence interval (CI) of all-cause mortality and tumor-specific mortality, respectively.

**Results:**

A total of 5,640 primary bone tumors were included in the study. In 45–59 years cohort, married, unmarried, divorced and widowed accounted for 66.0, 21.0, 11.2, and 1.8%, respectively; while 64.3, 10.1, 8.8, and 16.8% in 60+ years cohort, respectively. The widowed patients had a lower proportion of early-stage tumors at diagnosis than that married, unmarried, and divorced patients (31.0% vs. 36% vs. 37.1% vs. 39.4%; *P* = 0.008), and had a higher proportion of patients who did not undergo surgery than that of married, unmarried, and divorced patients (38.6% vs. 21.3% vs. 24.6% vs. 24.4%; *P* < 0.001). The widowed population had an increased risk of all-cause mortality (HR, 1.68; 95% CI, 1.50–1.88; *P* < 0.001) and disease-related mortality (HR, 1.33; 95% CI, 1.09–1.61; *P* = 0.005) compared with the married population.

**Conclusion:**

The marital status of middle-aged and elderly people can affect the tumor stage at diagnosis, treatment, and survival prognosis of patients with primary bone cancer. Widowed patients are more inclined to choose non-surgical treatment and have the worst prognosis.

## Introduction

Primary malignant bone tumors are uncommon in clinical settings and have low incidence (1). In 2022, the number of new primary malignant bone tumor cases is estimated to be 3,910 (1). A good prognosis of primary malignant bone tumors can be achieved through surgery and adjuvant radiotherapy and chemotherapy, and the 5-year survival rate can reach up to 70% (2–5). The prognosis of primary bone tumors is related to the age, histological tumor type, tumor stage, and other factors of patients (4, 6–8).

Studies have found that marital status is closely related to the survival outcomes of patients in many diseases, including some cancers (9–12). Married patients have a greater degree of social support than unmarried and widowed patients, which affects their overall health. A spouse not only offers material support in life but also provides tremendous emotional and psychological support during the most difficult period in the life of a patient, facilitates the access of a patient to key health services and plays an important role in the development of the entire condition for the better effect. For patients with bone tumors, surgery may interfere with their normal daily life. For example, patients who have undergone amputation cannot take care of themselves, and their spouses can provide great help to them. In a study, married patients with cancer had a lower risk of being diagnosed with cancer at an advanced stage than unmarried patients with cancer (12). In addition, married patients are more likely to receive appropriate treatment. At present, less attention is paid to the relationship between marital status and the prognosis of patients with primary bone cancer. Whether the marital status of patients with primary bone tumors is related to early diagnosis and treatment warrants further investigation.

Due to the rapid transformation of social economy and population structure, the distribution of marital status of middle-aged and elderly people is diversified, such as married, unmarried, divorced and widowed, which was related to family composition and living arrangements. Compared with the marital status of young people, middle-aged and elderly people may encounter difficulties in life, which are likely to be talking to each other and getting comfort from their spouses, while young people can also get help from their parents and get comfort from their hearts. A stable marriage is more likely to maintain a healthy lifestyle and positively face life’s setbacks. Therefore, the impact of marital status on the health status of middle-aged and elderly people is a topic worth exploring. In addition, considering the low incidence of primary bone tumors in middle-aged and elderly patients, less attention is currently paid to the effect of marital status on the treatment and prognosis of primary bone malignancies in middle-aged and elderly patients. In this study, the Surveillance, Epidemiology, and End Results (SEER) database was used to analyze the relationship between marital status and the treatment, staging and prognosis of middle-aged and elderly patients with primary bone tumors.

## Patients and methods

### Design and data sources

This retrospective observational cohort study was based on the Surveillance, Epidemiology, and End Results (SEER) database, which contains data of approximately 28% of the American population, with clinical and demographic characteristics comparable to those of the general population.^1^ The SEER program is a comprehensive data source widely used to study the incidence, staging, treatment, and survival rate of cancer. It reduces monitoring deviation caused by systematic, standardized, and regular data collection procedures to ensure quality assurance. The studies involving human participants were reviewed and approved by Fifth hospital in Wuhan. The SEER*Stat (version 8.4.0) software was used for downloading and analyzing the data.

### Population identification

The data of patients diagnosed with primary malignant bone tumors from 2000 to 2018 were extracted from the SEER database. The main inclusion criteria were as follows: (1) primary bone malignant confirmed *via* pathological examination based on the histologic type ICD-O-3 record; (2) age ≥45 years; (3) complete marital status information. Cases with larger missing information were not included for analysis.

### Study variables

The following demographic, clinical and pathological features were selected for analysis: marital status (married, unmarried, divorced and widowed), diagnostic years (2000–2003, 2004–2008, 2009–2013 and 2014–2018), diagnosis age (45–59 years and ≥ 60 years), sex (men and women), race (white, black and others [American Indian/Alaska natives, Asian natives and Asian/Pacific islanders]), median household income ($0–49,999; $50,000–59,999; $60,000–69,999 and > $70,000), residence (metropolitan area and non-metropolitan area), tumor site (limb, pelvic/spine and others), tumor histology (chondrosarcoma, osteosarcoma, and others), tumor grading (G1/G2, G3, and unknown); tumor size (0–5 cm, 5.1–10 cm, > 10 cm, and unknown), history of cancer (present or absent), surgical methods (no surgery, amputation, local/partial excision/destruction, radical excision with limb salvage, other/unknown), radiotherapy (with or without), chemotherapy (with or without/unknown), T staging (T1, T2, T3/4, Tx, and unknown), N staging (N0, N1, NX, and unknown) and M staging (M0, M1, MX, and unknown).

### Outcomes

In this study, the main endpoints were overall survival (OS) and cancer-specific survival (CSS). The detailed data regarding causes of death in each case were obtained from the SEER database. Death owing to primary bone cancer was considered special death from diseases, and the data were used to calculate CSS, whereas the data of all-cause death were used to calculate OS. Survival interval was defined as the time from the date of diagnosis of primary bone tumors to the date of death or loss to follow-up.

### Study objectives

The main purpose of this study was to examine whether marital status has an impact on the prognosis of middle-aged and elderly patients with bone tumors and is related to their treatment choice, tumor stage, and early death.

### Statistical analyses

All variables were defined as categorical variables, which were expressed as frequencies (%) and compared *via* the chi-square test. OS and CSS were compared among different subgroups *via* Kaplan–Meier method with the log-rank test. Cox proportional hazards regression model with a hazard ratio (HR) and Fine-and-Gray regression analysis with a sub-distribution hazard ratio (sHR) were used to evaluate the risk factors of overall mortality, cancer-specific mortality and other causes of mortality. The 95% confidence interval (CI) was also calculated along with HRs or sHRs. Logistic regression analysis was used to analyze the association of marital status with the surgical treatment choice, tumor stage and early death of middle-aged and elderly patients with bone tumors. The R statistical package (version 4.2.0; R Foundation for Statistical Computing, Vienna, Austria)^2^ was used to conduct all statistical analyses. All *P*-values were two-sided, with *P*-values of <0.05 indicating statistical significance.

## Results

### Baseline characteristics

Table 1 shows the demographic characteristics, clinicopathological features, and treatment information of patients with bone tumors. With an increase in diagnosis years, the number of patients recorded in the database gradually increased, from 17.7% in 2000–2004 to 30.3% in 2014–2018. The proportion of elderly patients (≥60 years) was higher than that of middle-aged patients (45–60 years) (57.9 vs. 42.1%, respectively), with the proportion of male patients being higher than that of female patients (54.7 vs. 45.3%, respectively). The proportion of patients with a high median household income and living in metropolitan areas was higher than those with a low median household income and living in non-metropolitan areas. Bone tumors of the trunk and limbs were most common (39.3%), followed by pelvic/spinal bone tumors (31.3%), and chondrosarcoma was the most common bone tumor in elderly patients (42.3%). Most patients received surgical treatment (76.1%), including amputation (7.3%), local/partial excision/destruction (29.4%), and radical excision with limb salvage (33.77%). Some patients received adjuvant radiotherapy (27.5%) and chemotherapy (22.1%).

**TABLE 1:** Demographic and clinic characteristics for patients with bone cancer as the primary malignant tumor between 2000 and 2018.

Characteristics	All	By marital status				
		
		Married	Unmarried	Divorced	Widowed	*P*-value
N	5,640	3,668 (65.0%)	828 (14.7%)	553 (9.8%)	591 (10.5%)	
Year at diagnosis. N (%)						<0.001
2000–2003	996 (17.7%)	692 (18.9%)	103 (12.4%)	84 (15.2%)	117 (19.8%)	
2004–2008	1,370 (24.3%)	865 (23.6%)	210 (25.4%)	127 (23.0%)	168 (28.4%)	
2009–2013	1,565 (27.7%)	1,001 (27.3%)	242 (29.2%)	169 (30.6%)	153 (25.9%)	
2014–2018	1,709 (30.3%)	1,110 (30.3%)	273 (33.0%)	173 (31.3%)	153 (25.9%)	
Age at diagnosis. Mean (SD)	63.6 (12.1)	62.9 (11.5)	58.6 (10.7)	61.5 (10.4)	76.6 (10.3)	<0.001
Age at diagnosis. N (%)						<0.001
45–59	2,375 (42.1%)	1,568 (42.7%)	499 (60.3%)	265 (47.9%)	43 (7.28%)	
60+	3,265 (57.9%)	2,100 (57.3%)	329 (39.7%)	288 (52.1%)	548 (92.7%)	
Sex. N (%)						<0.001
Female	2,555 (45.3%)	1,418 (38.7%)	380 (45.9%)	327 (59.1%)	430 (72.8%)	
Male	3,085 (54.7%)	2,250 (61.3%)	448 (54.1%)	226 (40.9%)	161 (27.2%)	
Race. N (%)						<0.001
White	4,822 (85.5%)	3,204 (87.4%)	659 (79.6%)	460 (83.2%)	499 (84.4%)	
Black	434 (7.70%)	201 (5.48%)	122 (14.7%)	61 (11.0%)	50 (8.46%)	
Other	384 (6.81%)	263 (7.17%)	47 (5.68%)	32 (5.79%)	42 (7.11%)	
Median household income. N (%)						0.019
0–$49,999	700 (12.4%)	457 (12.5%)	99 (12.0%)	68 (12.3%)	76 (12.9%)	
50,000–$59,999	802 (14.2%)	527 (14.4%)	128 (15.5%)	71 (12.8%)	76 (12.9%)	
60,000–$69,999	1,718 (30.5%)	1,073 (29.3%)	293 (35.4%)	179 (32.4%)	173 (29.3%)	
70,000+	2,420 (42.9%)	1,611 (43.9%)	308 (37.2%)	235 (42.5%)	266 (45.0%)	
Residence. N (%)						0.023
Metropolitan areas	5,020 (89.0%)	3,233 (88.1%)	757 (91.4%)	494 (89.3%)	536 (90.7%)	
Non-metropolitan areas	620 (11.0%)	435 (11.9%)	71 (8.57%)	59 (10.7%)	55 (9.31%)	
Site of tumor. N (%)						0.131
Limb	2,217 (39.3%)	1,421 (38.7%)	309 (37.3%)	234 (42.3%)	253 (42.8%)	
Pelvic/spin	1,765 (31.3%)	1,167 (31.8%)	251 (30.3%)	166 (30.0%)	181 (30.6%)	
Other	1,658 (29.4%)	1,080 (29.4%)	268 (32.4%)	153 (27.7%)	157 (26.6%)	
Grade of tumor. N (%)						0.003
G1/G2	1,983 (35.2%)	1,342 (36.6%)	286 (34.5%)	171 (30.9%)	184 (31.1%)	
G3	1,704 (30.2%)	1,112 (30.3%)	227 (27.4%)	186 (33.6%)	179 (30.3%)	
Unknown	1,953 (34.6%)	1,214 (33.1%)	315 (38.0%)	196 (35.4%)	228 (38.6%)	
Histology of tumor. N (%)						0.011
Chondrosarcoma	2,384 (42.3%)	1,620 (44.2%)	327 (39.5%)	215 (38.9%)	222 (37.6%)	
Osteosarcoma	1,076 (19.1%)	683 (18.6%)	166 (20.0%)	111 (20.1%)	116 (19.6%)	
Other	2,180 (38.7%)	1,365 (37.2%)	335 (40.5%)	227 (41.0%)	253 (42.8%)	
Size of tumor. N (%)						0.009
0–5 cm	1,326 (23.5%)	875 (23.9%)	200 (24.2%)	135 (24.4%)	116 (19.6%)	
5.1–10 cm	1,332 (23.6%)	838 (22.8%)	215 (26.0%)	145 (26.2%)	134 (22.7%)	
10+ cm	931 (16.5%)	602 (16.4%)	150 (18.1%)	86 (15.6%)	93 (15.7%)	
Unknown	2,051 (36.4%)	1,353 (36.9%)	263 (31.8%)	187 (33.8%)	248 (42.0%)	
Had prior tumor history. N (%)						<0.001
Yes	4,468 (79.2%)	2,913 (79.4%)	700 (84.5%)	435 (78.7%)	420 (71.1%)	
No	1,172 (20.8%)	755 (20.6%)	128 (15.5%)	118 (21.3%)	171 (28.9%)	
Surgery. N (%)						<0.001
None	1,327 (23.5%)	770 (21.0%)	198 (23.9%)	134 (24.2%)	225 (38.1%)	
Amputation	412 (7.30%)	254 (6.92%)	69 (8.33%)	53 (9.58%)	36 (6.09%)	
Local/partial excision/destruction	1,657 (29.4%)	1,115 (30.4%)	248 (30.0%)	150 (27.1%)	144 (24.4%)	
Radical excision with limb salvage	1,898 (33.7%)	1,294 (35.3%)	268 (32.4%)	176 (31.8%)	160 (27.1%)	
Other/unknown	346 (6.13%)	235 (6.41%)	45 (5.43%)	40 (7.23%)	26 (4.40%)	
Surgery. N (%)						<0.001
None	1,348 (23.9%)	782 (21.3%)	202 (24.4%)	136 (24.6%)	228 (38.6%)	
Had a surgical treatment	4,292 (76.1%)	2,886 (78.7%)	626 (75.6%)	417 (75.4%)	363 (61.4%)	
Radiation therapy. N (%)						0.075
None	4,087 (72.5%)	2,670 (72.8%)	618 (74.6%)	393 (71.1%)	406 (68.7%)	
Yes	1,553 (27.5%)	998 (27.2%)	210 (25.4%)	160 (28.9%)	185 (31.3%)	
Chemotherapy therapy. N (%)						<0.001
None	4,391 (77.9%)	2,823 (77.0%)	640 (77.3%)	417 (75.4%)	511 (86.5%)	
Yes	1,249 (22.1%)	845 (23.0%)	188 (22.7%)	136 (24.6%)	80 (13.5%)	
T stage. N (%)						0.008
T1	2,030 (36.0%)	1,322 (36.0%)	307 (37.1%)	218 (39.4%)	183 (31.0%)	
T2	1,252 (22.2%)	806 (22.0%)	205 (24.8%)	124 (22.4%)	117 (19.8%)	
T3/T4	159 (2.82%)	104 (2.84%)	19 (2.29%)	10 (1.81%)	26 (4.40%)	
TX	885 (15.7%)	574 (15.6%)	123 (14.9%)	82 (14.8%)	106 (17.9%)	
Unknown	1,314 (23.3%)	862 (23.5%)	174 (21.0%)	119 (21.5%)	159 (26.9%)	
N stage. N (%)						0.076
N0	3,924 (69.6%)	2,554 (69.6%)	592 (71.5%)	400 (72.3%)	378 (64.0%)	
N1	104 (1.84%)	67 (1.83%)	18 (2.17%)	7 (1.27%)	12 (2.03%)	
NX	298 (5.28%)	185 (5.04%)	44 (5.31%)	27 (4.88%)	42 (7.11%)	
Unknown	1,314 (23.3%)	862 (23.5%)	174 (21.0%)	119 (21.5%)	159 (26.9%)	
M stage. N (%)						0.053
M0	3,672 (65.1%)	2,371 (64.6%)	574 (69.3%)	372 (67.3%)	355 (60.1%)	
M1	575 (10.2%)	387 (10.6%)	67 (8.09%)	54 (9.76%)	67 (11.3%)	
MX	79 (1.40%)	48 (1.31%)	13 (1.57%)	8 (1.45%)	10 (1.69%)	
Unknown	1,314 (23.3%)	862 (23.5%)	174 (21.0%)	119 (21.5%)	159 (26.9%)	

Most middle-aged and elderly patients with bone tumors were married (65%). Unmarried, divorced, and widowed patients accounted for 14.7, 9.8, and 10.5%, respectively. Married, unmarried, divorced and widowed 45–59 years patients accounted for 66.0, 21.0, 11.2, and 1.8%, respectively; while for it accounted for 64.3, 10.1, 8.8, and 16.8% for 60+ years patients, respectively (data not shown). The proportion of widowed men was lower than that of widowed women (5.2 vs. 16.8%, respectively; Supplementary Table 1). The proportion of elderly widowed patients aged > 60 years was higher than that of middle-aged widowed patients (92.7 vs. 7.8%, respectively). The widowed patients had a lower proportion of T1 stage tumors at diagnosis than that of married, unmarried, and divorced patients (31.0% vs. 36% vs. 37.1% vs. 39.4%; *P* = 0.008), and widowed patients had a higher proportion of patients who did not undergo surgery than that of married, unmarried, and divorced patients (38.6% vs. 21.3% vs. 24.6% vs. 24.4%; *P* < 0.001). Among widowed patients, the proportion of patients who received chemotherapy was lower than that of married, unmarried, and divorced patients (13.5% vs. 23.0%, 22.7% and 24.6%, respectively). Besides, among widowed patients, the proportion of patients with T3/4-, N1-, and M1-stage disease was higher than that of married, unmarried, and divorced patients.

### Survival outcomes

**FIGURE 1:**
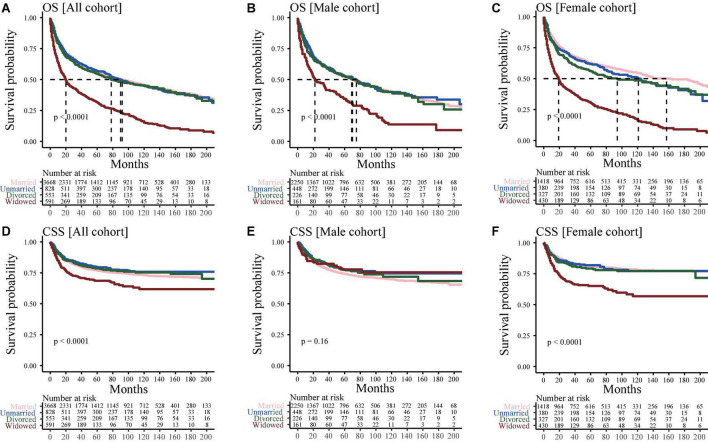
Kaplan–Meier curve for analyzing the effects of marital status on survival. **(A)** Overall survival (OS) in all cohorts; **(B)** OS in the male cohort; **(C)** OS in the female cohort; **(D)** Cancer-special survival (CSS) in all cohorts **(E)** CSS in the male cohort; **(F)** CSS in the female cohort.

Figure 1 shows the OS and CSS of different marital status groups. Widowed patients with bone tumors had the worst survival outcome, with a median OS of only 20 months in the widowed group as compared with the median OS of 92, 90, and 78 months in the married, unmarried, and divorced groups, respectively (Figure 1A); while, in Figures 1B,C showed a similar result. In all cohort, widowed patients with bone tumors had the worst CSS (Figure 1D). There was no difference was observed in CSS between widowed male patients and married, unmarried, and divorced male patients (Figure 1E). However, in female cohort, widowed patients had a worse CSS than married, unmarried and divorced patients (Figure 1F).

Since the older the patients, the greater the probability of widowhood. Patients were divided into two groups based on their age: middle-aged (45–59 years) and elderly (≥60 years) groups. Supplementary Figure 1A showed that the median OS of middle-aged widowed, married, unmarried and divorced patients was 110, 179, 138, and 187 months, respectively, whereas that of elderly widowed, married, unmarried and divorced patients was 19, 50, 57, and 41 months, respectively (Supplementary Figure 1B). However, for middle-aged patients, there was no difference was observed in CSS between widowed male patients and married, unmarried and divorced male patients (Supplementary Figure 1C); while, elderly widowed still had a worse CSS than married, unmarried and divorced patients (Supplementary Figure 1B). In addition, after adjusting for variables of age and others, consistent results were found (Supplementary Figure 1).

**FIGURE 2:**
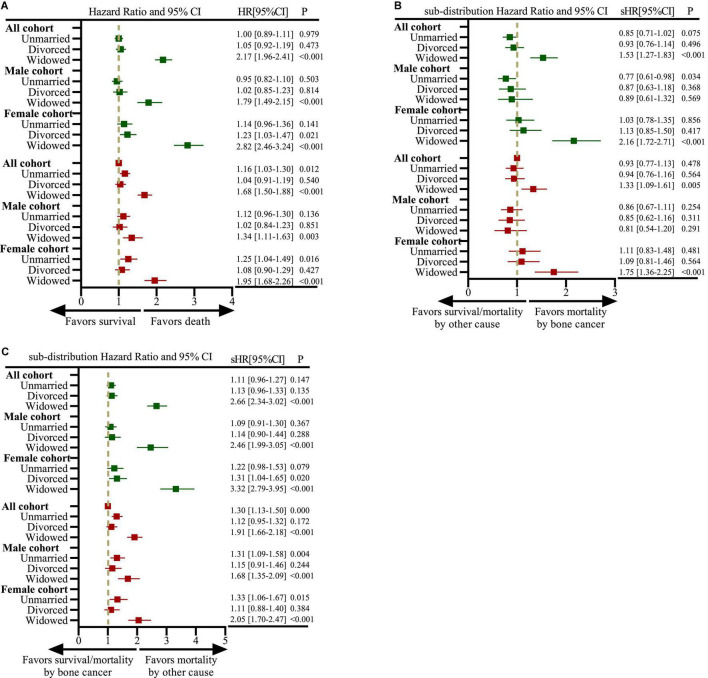
Forest plots depicting hazard ratios (HRs) and 95% confidence intervals (CIs) for analyzing the impact of marital status on all-cause mortality based on the Cox proportional hazards regression model and mortality owing to bone cancer and other cancers based on the Fine-and-Gray competing risk model. **(A)** Impact of marital status on all-cause mortality in all, male and female cohorts; **(B)** impact of marital status on mortality owing to bone cancer in all, male and female cohorts; **(C)** impact of marital status on mortality owing to other cancers in all, male and female cohorts. The dark green color represents the results of univariate analysis, and the brown color represents the results of multivariate analysis.

Figure 2 showed that multivariate analysis revealed that the risk of all-cause mortality (HR: 1.68, 95%CI, 1.50–1.88, *P* < 0.001) and competitive non-cancer-specific mortality (sHR: 1.91, 95%CI, 1.66–2.18, *P* < 0.001) was higher in the widowed population than in the married population; however, the risk of disease-specific death risk among the widowed and married male patients showed no significant (sHR: 0.81, 95%CI, 0.54–1.20, *P* = 0.291).

Given tumor histology and stage at diagnosis were two important prognosis factors in the present study. In different histological subgroups, the marital status of patients can affect OS and CSS outcomes (Supplementary Figure 2), but after adjusting for age and other factors, it was found that OS and CSS of widowed patients were worse than those of other marital statuses only in chondrosarcoma patients (data not shown). While the marital status had different effects on OS and CSS among different tumor stage subgroups (Supplementary Figure 3). For patients in local and regional subgroups, widowed patients had the worst OS, however, after adjusting for age and other factors, marital status was not associated with OS or CSS in metastatic subgroups (data not shown).

### Impact of marital status on treatment, tumor stage, and early death

**FIGURE 3:**
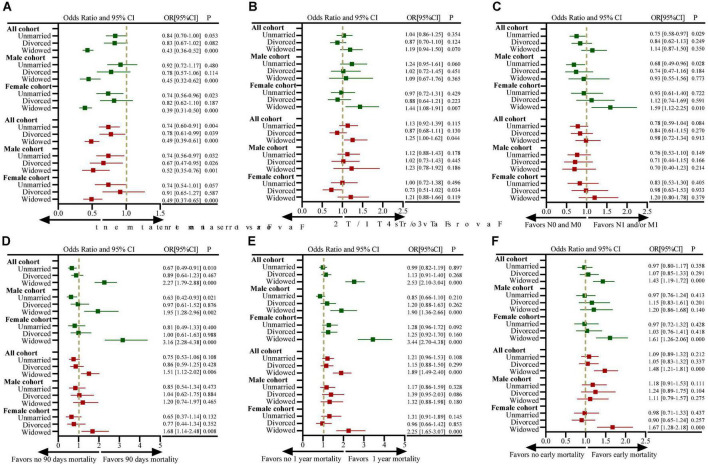
Forest plots depicting odds ratios (ORs) and 95% confidence intervals (CIs) for examining the association between marital status and **(A)** the use of definitive surgical treatment (vs. no use of surgical treatment); **(B)** presentation with T3/T4-stage disease (vs. T1/T2-stage disease); **(C)** presentation with N1- and/or M1-stage disease (vs. N0- and M0-stage disease); **(D)** 90-day mortality (vs. > 90-day mortality); **(E)** 1-year mortality (vs. > 1-year mortality); **(F)** early mortality. ORs for the measure of use of definitive surgical treatment are adjusted for covariables of year at diagnosis, age at diagnosis, sex, race, median household income, residence, site of tumors, tumor grade, histological features, previous tumor history, T staging, N staging and M staging; ORs for the measure of TNM staging are adjusted for covariables of year at diagnosis, age at diagnosis, sex, race, median household income, residence, site of tumors, tumor grade, histological features, previous tumor history (all analyses were based on the SEER data, excluding cases with missing TNM stage information); ORs for the measure of 90-day and 1-year mortality are adjusted for covariables of year at diagnosis, age at diagnosis, sex, race, median household income, residence, site of tumors, tumor grade, histological features, previous tumor history, T staging, N staging, M staging, surgical treatment, radiation therapy, and chemotherapy; ORs for the measure of early mortality using ordered logistic regression analysis are adjusted for covariables of year at diagnosis, age at diagnosis, sex, race, median household income, residence, site of tumors, tumor grade, histological features, previous tumor history, T staging, N staging, M staging, surgical treatment, radiation therapy and chemotherapy. The “sex” variable was not included in the analysis of male and female subgroups. The dark green color represents the results of univariate analysis, and the brown color represents the results of multivariate analysis.

Figure 3 shows the impact of marital status on the treatment, tumor stage and early death of all, male and female patients. Widowed patients were more likely not undergo tumor-related surgical treatment compared with married patients (OR: 0.43, 95%CI, 0.36–0.52, *P* < 0.001; Figure 3A). Widowed patients have more proportion of T3/T4, N1/M1 stage disease at diagnosis than married patients (Table 1), in addition, it was statistical association with T3/T4 stage after adjusting for other factors (OR: 1.25, 95%CI, 1.00–1.62, *P* = 0.044; Figure 3B). However, no statistical correlation was found between widowed patients and N1/M1 stage at diagnosis of primary bone tumor (OR: 0.98, 95%CI, 0.40–1.34, *P* = 0.913; Figure 3C). Furthermore, female widowed patients were more likely to die early after the diagnosis and treatment of the disease, with an increased risk of 90-day and 1-year all-cause mortality (Figures 3D–F).

## Discussion

To develop more individualized and holistic treatment strategies for primary bone malignancies, it is necessary to elucidate the impact of marital status on survival. In this study, we assessed the significance of marital status (married, unmarried, divorced and widowed) on the survival outcomes of patients with primary bone malignancies based on a population-based comprehensive analysis. Different marital subgroups showed different OS and CSS performances. Univariate and multivariate Cox and Fine-and-Gray regression analyses showed that widowhood was an independent predictor of all-cause and disease-specific mortality risks. In addition, marital status was associated with OS but not with CSS among men; however, it was associated with both OS and CSS among women. Therefore, marital status is of great significance to the survival outcomes of patients with primary bone tumors.

In most cases, widowed patients had the worst survival outcome. Many recent studies have reported that marital status can be used as an independent factor to predict the survival rate of patients with different tumors. For example, Wang et al. (13) examined the relationship between marital status and survival based on epithelial ovarian cancer data obtained from the SEER database and found that marital status was an independent predictor of OS and CSS in ovarian cancer. In a study of patients with esophageal cancer (14), the unmarried, divorced/separated and widowed groups had a higher risk of death in all aspects than the married group. In another population-based astrocytoma study, Xie et al. (15) showed that married patients had the highest OS and CSS, whereas divorced/separated or widowed patients had worse CSS. Many recent studies have reported similar results on several other cancers, such as chondrosarcoma (16), thyroid cancer (17), gastric cancer (18), breast cancer (19), testicular cancer (20), and cutaneous melanoma (21).

Marital status may affect the survival of patients in several ways. Because marriage is the main source of social support, patients are more likely to seek prompt medical treatment (22). Individuals who report higher satisfaction with social support are at lower risk than those who report lower satisfaction (23). Spousal support can increase the convenience of medical screening, adherence to prescribed treatment regimens and the likelihood of receiving more aggressive treatment (24–26). Married patients can receive support and encouragement from their spouses to actively visit the hospital for examination (27). Delayed diagnosis may also affect the survival of patients. Furthermore, tumor size, grade and stage have been identified as important factors for predicting the survival of patients with bone tumors (28, 29). In the present study, female widowed patients had the largest tumor size and the highest prevalence of regional and distant metastases, which may account for the low survival rate owing to delayed diagnosis. A high rate of delayed diagnosis among widowed patients has also been observed in other studies. For example, Shi et al. (17) found that widowed patients with differentiated thyroid cancer were at the most advanced tumor stage and had the highest prevalence of distant metastasis. In addition, lower socioeconomic status has been identified as a risk factor for predicting the survival of patients with multiple myeloma (30) and testicular germ cell tumors (31). Financial assistance can relieve many non-medical-related pressures, thus enabling patients to avail more advanced medical facilities, adopt a better lifestyle and attain a higher standard of living (32). Therefore, low socioeconomic status may be another reason for the poor prognosis of widowed patients. However, in this study, relevant information in this regard could not be obtained because the data were extracted from a database. In addition to the abovementioned reasons, psychological burden may also play an important role in predicting the survival of patients with cancer. Married patients can share their emotional burden with their spouses, which can improve their survival outcomes (33, 34). Furthermore, the association between poor survival and widowhood can be hypothetically explained based on psychosocial factors. The death of a spouse can be very stressful for their partner because they must transition and adjust to a new social role. Therefore, widowhood is associated with a higher risk of psychological disorders, and owing to the lack of advantages of emotional, psychological, and psychosocial support, widowed patients are predisposed to poor survival outcomes.

In this study, the proportion of middle-aged and elderly widowed patients was as high as 92.7%, which was significantly higher than that of the married, unmarried, and divorced patients. Elderly patients are more likely to die owing to poor physical fitness and more complications (35), which may be one of the important reasons for the low survival rate in the widowed group. In addition, the widowed group had the highest proportion of women (72.8%). An interesting study found that widowed women had lower natural killer cell activity and higher plasma cortisol levels than the control population, which may be associated with increased mortality among widowed patients (36). In another study, widowed patients had the highest non-surgical rate (38.6%), and inadequate treatment led to a worse prognosis in the widowed group (14). In this study, after adjusting for other confounding factors, a significant association was observed between widowhood and non-surgical treatment. A study on gastric cancer reported that inadequate social support owing to the lack of spousal support was attributed to the lowest rate of surgery in widowed patients (37). In addition, widowed patients have an increased risk of stress and psychiatric disorders owing to a lack of a partner (15). However, married patients have better family conditions and receive more social support from their spouses and family members (38). Therefore, a good marital status can help patients in reducing anxiety, stress, depression, and other negative emotions and receiving more material support.

However, some studies have suggested that marital status is not significantly associated with the prognosis of cancer. Goodwin et al. (39) recognized the limitations of not being able to analyze and control the socioeconomic status of patients in a database and suggested that marital status did not affect the survival of patients with epithelial cancer. Jatoi et al. used the database of Mayo Clinic and reported that no significant difference in survival was observed among patients with non-small cell lung cancer with different marital statuses, which is inconsistent with the findings of this study (40, 41). Jatoi et al. suggested that patients with strong social support and high socioeconomic status skewed the data, thus explaining conflicting results (42). Therefore, further investigation is warranted to verify the benefits of marriage among patients with different cancer types. In addition, we found that marital status does not have an impact on the prognosis of all primary bone cancers. For example, we found that for patients with metastatic disease at the time of diagnosis, the oncological characteristics of the disease itself may be the most important prognostic factor, and the effect of marital status is weakened.

This study has several limitations. Because this was a retrospective study, selection bias is inevitable. Therefore, prospective studies are required to verify the results. Additionally, the SEER database only provides data regarding marital status at diagnosis; marital status might have changed during treatment, which may affect the findings of this study. Besides, we cannot obtain whether the patients in different marital statuses are separated and the relationship between the husband and wife through the SEER database, which will also bring different psychological effects to the patients. There are some comorbidities in middle-aged and elderly patients. These factors are important risk factors for the prognosis of cancer patients. However, the SEER database does not record the patient’s comorbidities and complications, and these factors have not been included in the multivariate analysis. In addition, we speculated that psychological factors and socioeconomic status are the main reasons for the poor prognosis of widowed patients; however, the SEER database neither allowed rigorous psychosocial testing nor had records related to the socioeconomic status of patients to test this hypothesis. Therefore, further clinical, and psychological studies and socioeconomic status profiling are required to validate the findings of this study.

## Conclusion

The survival of middle-aged and elderly patients with primary bone cancer was significantly correlated with their marital status. The poor prognosis of widowed patients can be attributed to the following reasons: delayed diagnosis of tumors; heavier tumor burden at the time of diagnosis and the increased risk of local and distant metastases, leading to death. In the future, more attention should be paid to the influence of marital status on the prognosis of patients with bone tumors. In particular, widowed patients should be provided with more health guidance to facilitate prompt diagnosis and surgical treatment, thereby reducing the incidence of early death.

## Data availability statement

The original contributions presented in this study are included in the article/Supplementary material, further inquiries can be directed to the corresponding author.

## Ethics statement

The studies involving human participants were reviewed and approved by the Fifth Hospital in Wuhan. The patients/participants provided their written informed consent to participate in this study.

## Author contributions

HZ, KZ, and YW conceived the research and wrote the manuscript. HZ, KZ, and ML analyzed the data and prepared the figures and tables. All authors were involved in revising the manuscript and have approved its final version.

## Footnotes

1
https://seer.cancer.gov/

2
https://www.r-project.org
