# Supplementary material for: Influence of marital status on the treatment and survival of middle-aged and elderly patients with primary bone cancer
Source: Front Med (Lausanne). 2022 Oct 18;9:1001522. doi: 10.3389/fmed.2022.1001522 (PMC9623305; doi:10.3389/fmed.2022.1001522)
Supplement: Supplementary file 1 [file Table_1.pdf]

Supplementary Table 1. Demographic and clinic characteristics for patients with bone cancer as the primary malignant tumor between 2000–2018.

Characteristics	Male cohort					Female cohort				
	All	By marital status				All	By marital status			
		Married	Unmarried	Divorced	Widowed		Married	Unmarried	Divorced	Widowed
**N**	3085	2250 (72.9%)	448 (14.5%)	226 (7.3%)	161 (5.2%)	2555	1418 (55.5%)	380 (14.9%)	327 (12.8%)	430 (16.8%)
**Year at diagnosis. N (%)**										
2000-2003	537 (17.4%)	419 (18.6%)	55 (12.3%)	30 (13.3%)	33 (20.5%)	459 (18.0%)	273 (19.3%)	48 (12.6%)	54 (16.5%)	84 (19.5%)
2004-2008	728 (23.6%)	533 (23.7%)	104 (23.2%)	43 (19.0%)	48 (29.8%)	642 (25.1%)	332 (23.4%)	106 (27.9%)	84 (25.7%)	120 (27.9%)
2009-2013	877 (28.4%)	624 (27.7%)	136 (30.4%)	75 (33.2%)	42 (26.1%)	688 (26.9%)	377 (26.6%)	106 (27.9%)	94 (28.7%)	111 (25.8%)
2014-2018	943 (30.6%)	674 (30.0%)	153 (34.2%)	78 (34.5%)	38 (23.6%)	766 (30.0%)	436 (30.7%)	120 (31.6%)	95 (29.1%)	115 (26.7%)
**Age at diagnosis. Mean (SD)**	63.5 (11.8)	64.0 (11.6)	57.8 (10.4)	60.5 (9.65)	76.4 (10.1)	63.7 (12.5)	61.2 (11.2)	59.5 (10.9)	62.2 (10.8)	76.7 (10.4)
**Age at diagnosis. N (%)**										
45-59	1292 (41.9%)	884 (39.3%)	283 (63.2%)	116 (51.3%)	9 (5.59%)	1083 (42.4%)	684 (48.2%)	216 (56.8%)	149 (45.6%)	34 (7.91%)
60+	1793 (58.1%)	1366 (60.7%)	165 (36.8%)	110 (48.7%)	152 (94.4%)	1472 (57.6%)	734 (51.8%)	164 (43.2%)	178 (54.4%)	396 (92.1%)
**Race. N (%)**										
White	2655 (86.1%)	1975 (87.8%)	356 (79.5%)	185 (81.9%)	139 (86.3%)	2167 (84.8%)	1229 (86.7%)	303 (79.7%)	275 (84.1%)	360 (83.7%)
Black	217 (7.03%)	122 (5.42%)	65 (14.5%)	21 (9.29%)	9 (5.59%)	217 (8.49%)	79 (5.57%)	57 (15.0%)	40 (12.2%)	41 (9.53%)
Other	213 (6.90%)	153 (6.80%)	27 (6.03%)	20 (8.85%)	13 (8.07%)	171 (6.69%)	110 (7.76%)	20 (5.26%)	12 (3.67%)	29 (6.74%)
**Median household income. N (%)**										
$0-$49,999	389 (12.6%)	277 (12.3%)	56 (12.5%)	37 (16.4%)	19 (11.8%)	311 (12.2%)	180 (12.7%)	43 (11.3%)	31 (9.48%)	57 (13.3%)
$50,000-$59,999	421 (13.6%)	307 (13.6%)	67 (15.0%)	25 (11.1%)	22 (13.7%)	381 (14.9%)	220 (15.5%)	61 (16.1%)	46 (14.1%)	54 (12.6%)
$60,000-$69,999	964 (31.2%)	694 (30.8%)	162 (36.2%)	67 (29.6%)	41 (25.5%)	754 (29.5%)	379 (26.7%)	131 (34.5%)	112 (34.3%)	132 (30.7%)
$70,000+	1311 (42.5%)	972 (43.2%)	163 (36.4%)	97 (42.9%)	79 (49.1%)	1109 (43.4%)	639 (45.1%)	145 (38.2%)	138 (42.2%)	187 (43.5%)
**Residence. N (%)**										
Metropolitan areas	2743 (88.9%)	1987 (88.3%)	411 (91.7%)	196 (86.7%)	149 (92.5%)	2277 (89.1%)	1246 (87.9%)	346 (91.1%)	298 (91.1%)	387 (90.0%)
Nonmetropolitan areas	342 (11.1%)	263 (11.7%)	37 (8.26%)	30 (13.3%)	12 (7.45%)	278 (10.9%)	172 (12.1%)	34 (8.95%)	29 (8.87%)	43 (10.0%)
**Site of tumor. N (%)**										
Limb	1157 (37.5%)	856 (38.0%)	159 (35.5%)	82 (36.3%)	60 (37.3%)	1060 (41.5%)	565 (39.8%)	150 (39.5%)	152 (46.5%)	193 (44.9%)
Pelvic/spin	1033 (33.5%)	755 (33.6%)	146 (32.6%)	73 (32.3%)	59 (36.6%)	732 (28.6%)	412 (29.1%)	105 (27.6%)	93 (28.4%)	122 (28.4%)
Other	895 (29.0%)	639 (28.4%)	143 (31.9%)	71 (31.4%)	42 (26.1%)	763 (29.9%)	441 (31.1%)	125 (32.9%)	82 (25.1%)	115 (26.7%)
**Grade of tumor. N (%)**										
G1/G2	1018 (33.0%)	755 (33.6%)	147 (32.8%)	66 (29.2%)	50 (31.1%)	965 (37.8%)	587 (41.4%)	139 (36.6%)	105 (32.1%)	134 (31.2%)
G3	985 (31.9%)	734 (32.6%)	128 (28.6%)	74 (32.7%)	49 (30.4%)	719 (28.1%)	378 (26.7%)	99 (26.1%)	112 (34.3%)	130 (30.2%)
Unknown	1082 (35.1%)	761 (33.8%)	173 (38.6%)	86 (38.1%)	62 (38.5%)	871 (34.1%)	453 (31.9%)	142 (37.4%)	110 (33.6%)	166 (38.6%)
**Histology of tumor. N (%)**										
Chondrosarcoma	1257 (40.7%)	960 (42.7%)	160 (35.7%)	83 (36.7%)	54 (33.5%)	1127 (44.1%)	660 (46.5%)	167 (43.9%)	132 (40.4%)	168 (39.1%)
Osteosarcoma	574 (18.6%)	413 (18.4%)	85 (19.0%)	45 (19.9%)	31 (19.3%)	502 (19.6%)	270 (19.0%)	81 (21.3%)	66 (20.2%)	85 (19.8%)
Other	1254 (40.6%)	877 (39.0%)	203 (45.3%)	98 (43.4%)	76 (47.2%)	926 (36.2%)	488 (34.4%)	132 (34.7%)	129 (39.4%)	177 (41.2%)
**Size of tumor. N (%)**										
0-5cm	674 (21.8%)	495 (22.0%)	97 (21.7%)	48 (21.2%)	34 (21.1%)	652 (25.5%)	380 (26.8%)	103 (27.1%)	87 (26.6%)	82 (19.1%)
5.1-10cm	740 (24.0%)	523 (23.2%)	127 (28.3%)	63 (27.9%)	27 (16.8%)	592 (23.2%)	315 (22.2%)	88 (23.2%)	82 (25.1%)	107 (24.9%)
10+cm	573 (18.6%)	412 (18.3%)	92 (20.5%)	43 (19.0%)	26 (16.1%)	358 (14.0%)	190 (13.4%)	58 (15.3%)	43 (13.1%)	67 (15.6%)

Unknown	1098 (35.6%)	820 (36.4%)	132 (29.5%)	72 (31.9%)	74 (46.0%)	953 (37.3%)	533 (37.6%)	131 (34.5%)	115 (35.2%)	174 (40.5%)
**Had prior tumor history. N (%)**										
Yes	2443 (79.2%)	1761 (78.3%)	384 (85.7%)	188 (83.2%)	110 (68.3%)	2025 (79.3%)	1152 (81.2%)	316 (83.2%)	247 (75.5%)	310 (72.1%)
No	642 (20.8%)	489 (21.7%)	64 (14.3%)	38 (16.8%)	51 (31.7%)	530 (20.7%)	266 (18.8%)	64 (16.8%)	80 (24.5%)	120 (27.9%)
**Surgery. N (%)**										
None	718 (23.3%)	493 (21.9%)	105 (23.4%)	59 (26.1%)	61 (37.9%)	609 (23.8%)	277 (19.5%)	93 (24.5%)	75 (22.9%)	164 (38.1%)
Amputation	248 (8.04%)	175 (7.78%)	42 (9.38%)	21 (9.29%)	10 (6.21%)	164 (6.42%)	79 (5.57%)	27 (7.11%)	32 (9.79%)	26 (6.05%)
Local/partial excision/destruction	853 (27.6%)	643 (28.6%)	123 (27.5%)	52 (23.0%)	35 (21.7%)	804 (31.5%)	472 (33.3%)	125 (32.9%)	98 (30.0%)	109 (25.3%)
Radical excision with limb salvage	1065 (34.5%)	789 (35.1%)	155 (34.6%)	76 (33.6%)	45 (28.0%)	833 (32.6%)	505 (35.6%)	113 (29.7%)	100 (30.6%)	115 (26.7%)
Other/unknown	201 (6.52%)	150 (6.67%)	23 (5.13%)	18 (7.96%)	10 (6.21%)	145 (5.68%)	85 (5.99%)	22 (5.79%)	22 (6.73%)	16 (3.72%)
**Surgery. N (%)**										
None	734 (23.8%)	503 (22.4%)	107 (23.9%)	61 (27.0%)	63 (39.1%)	614 (24.0%)	279 (19.7%)	95 (25.0%)	75 (22.9%)	165 (38.4%)
Had a surgical treatment	2351 (76.2%)	1747 (77.6%)	341 (76.1%)	165 (73.0%)	98 (60.9%)	1941 (76.0%)	1139 (80.3%)	285 (75.0%)	252 (77.1%)	265 (61.6%)
**Radiation therapy. N (%)**										
None	2222 (72.0%)	1622 (72.1%)	329 (73.4%)	157 (69.5%)	114 (70.8%)	1865 (73.0%)	1048 (73.9%)	289 (76.1%)	236 (72.2%)	292 (67.9%)
Yes	863 (28.0%)	628 (27.9%)	119 (26.6%)	69 (30.5%)	47 (29.2%)	690 (27.0%)	370 (26.1%)	91 (23.9%)	91 (27.8%)	138 (32.1%)
**Chemotherapy therapy. N (%)**										
None	2362 (76.6%)	1709 (76.0%)	339 (75.7%)	174 (77.0%)	140 (87.0%)	2029 (79.4%)	1114 (78.6%)	301 (79.2%)	243 (74.3%)	371 (86.3%)
Yes	723 (23.4%)	541 (24.0%)	109 (24.3%)	52 (23.0%)	21 (13.0%)	526 (20.6%)	304 (21.4%)	79 (20.8%)	84 (25.7%)	59 (13.7%)
**T stage. N (%)**										
T1	1032 (33.5%)	753 (33.5%)	155 (34.6%)	80 (35.4%)	44 (27.3%)	998 (39.1%)	569 (40.1%)	152 (40.0%)	138 (42.2%)	139 (32.3%)
T2	773 (25.1%)	550 (24.4%)	132 (29.5%)	63 (27.9%)	28 (17.4%)	479 (18.7%)	256 (18.1%)	73 (19.2%)	61 (18.7%)	89 (20.7%)
T3/T4	88 (2.85%)	61 (2.71%)	12 (2.68%)	5 (2.21%)	10 (6.21%)	71 (2.78%)	43 (3.03%)	7 (1.84%)	5 (1.53%)	16 (3.72%)
TX	468 (15.2%)	355 (15.8%)	49 (10.9%)	31 (13.7%)	33 (20.5%)	417 (16.3%)	219 (15.4%)	74 (19.5%)	51 (15.6%)	73 (17.0%)
Unknown	724 (23.5%)	531 (23.6%)	100 (22.3%)	47 (20.8%)	46 (28.6%)	590 (23.1%)	331 (23.3%)	74 (19.5%)	72 (22.0%)	113 (26.3%)
**N stage. N (%)**										
N0	2130 (69.0%)	1550 (68.9%)	323 (72.1%)	161 (71.2%)	96 (59.6%)	1794 (70.2%)	1004 (70.8%)	269 (70.8%)	239 (73.1%)	282 (65.6%)
N1	65 (2.11%)	47 (2.09%)	10 (2.23%)	5 (2.21%)	3 (1.86%)	39 (1.53%)	20 (1.41%)	8 (2.11%)	2 (0.61%)	9 (2.09%)
NX	166 (5.38%)	122 (5.42%)	15 (3.35%)	13 (5.75%)	16 (9.94%)	132 (5.17%)	63 (4.44%)	29 (7.63%)	14 (4.28%)	26 (6.05%)
Unknown	724 (23.5%)	531 (23.6%)	100 (22.3%)	47 (20.8%)	46 (28.6%)	590 (23.1%)	331 (23.3%)	74 (19.5%)	72 (22.0%)	113 (26.3%)
**M stage. N (%)**										
M0	1965 (63.7%)	1412 (62.8%)	307 (68.5%)	153 (67.7%)	93 (57.8%)	1707 (66.8%)	959 (67.6%)	267 (70.3%)	219 (67.0%)	262 (60.9%)
M1	354 (11.5%)	277 (12.3%)	38 (8.48%)	22 (9.73%)	17 (10.6%)	221 (8.65%)	110 (7.76%)	29 (7.63%)	32 (9.79%)	50 (11.6%)
MX	42 (1.36%)	30 (1.33%)	3 (0.67%)	4 (1.77%)	5 (3.11%)	37 (1.45%)	18 (1.27%)	10 (2.63%)	4 (1.22%)	5 (1.16%)
Unknown	724 (23.5%)	531 (23.6%)	100 (22.3%)	47 (20.8%)	46 (28.6%)	590 (23.1%)	331 (23.3%)	74 (19.5%)	72 (22.0%)	113 (26.3%)
