# Supplementary material for: Influence of marital status on the treatment and survival of middle-aged and elderly patients with primary bone cancer
Source: Front Med (Lausanne). 2022 Oct 18;9:1001522. doi: 10.3389/fmed.2022.1001522 (PMC9623305; doi:10.3389/fmed.2022.1001522)
Supplement: Supplementary file 2 [file Image_1.pdf]

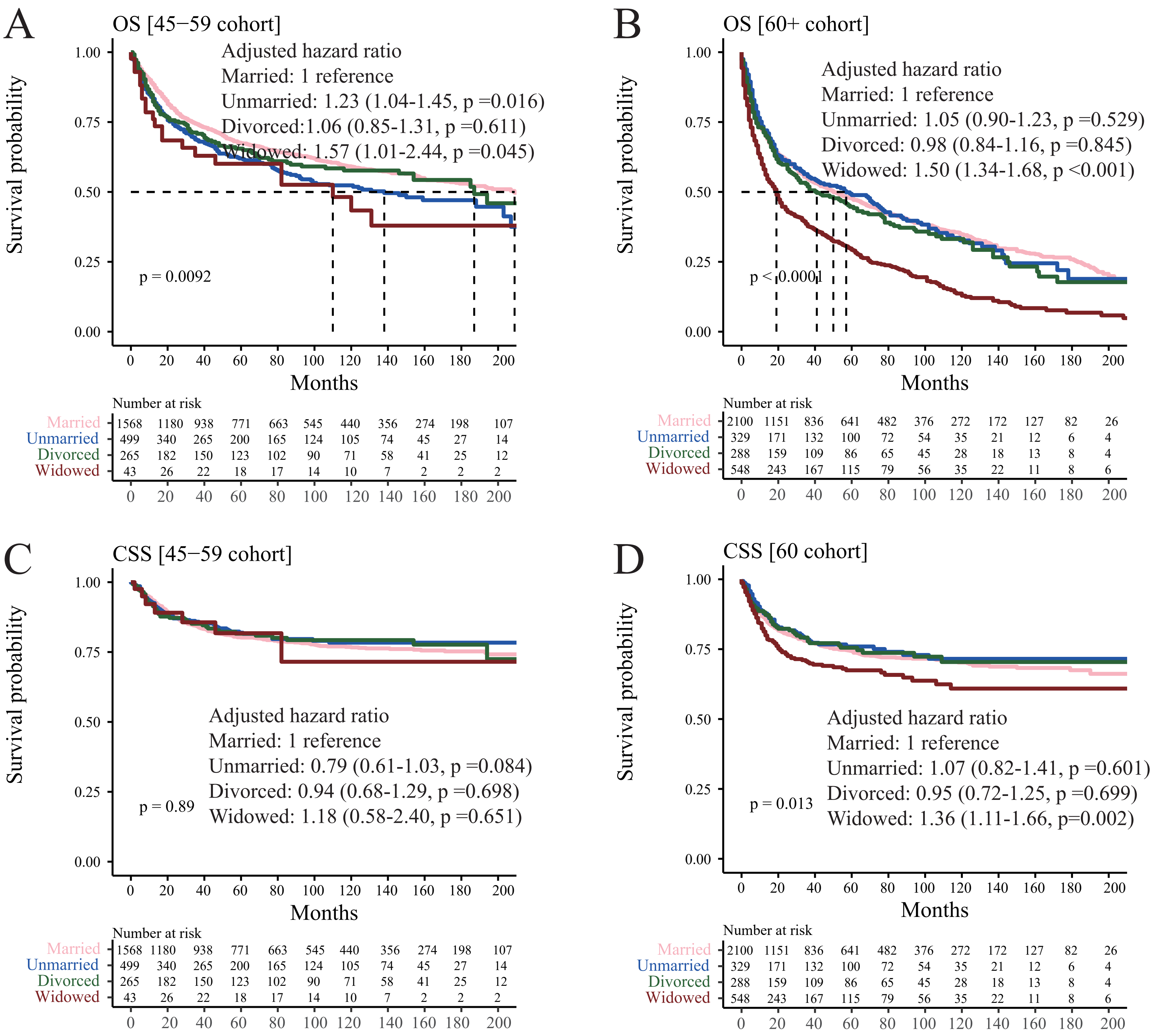


**Supplementary Figure 1.** Kaplan–Meier curve for analysing the effects of marital status on survival. (A) Overall survival (OS) in the middle-aged cohort (age, 45–59 years); (B) OS in the elderly cohort (age, ≥60 years); (C) Cancer-special survival (CSS) in the middle-aged cohort; (D) CSS in the elderly cohort.
