# Supplementary material for: Influence of marital status on the treatment and survival of middle-aged and elderly patients with primary bone cancer
Source: Front Med (Lausanne). 2022 Oct 18;9:1001522. doi: 10.3389/fmed.2022.1001522 (PMC9623305; doi:10.3389/fmed.2022.1001522)
Supplement: Supplementary file 4 [file Image_3.pdf]

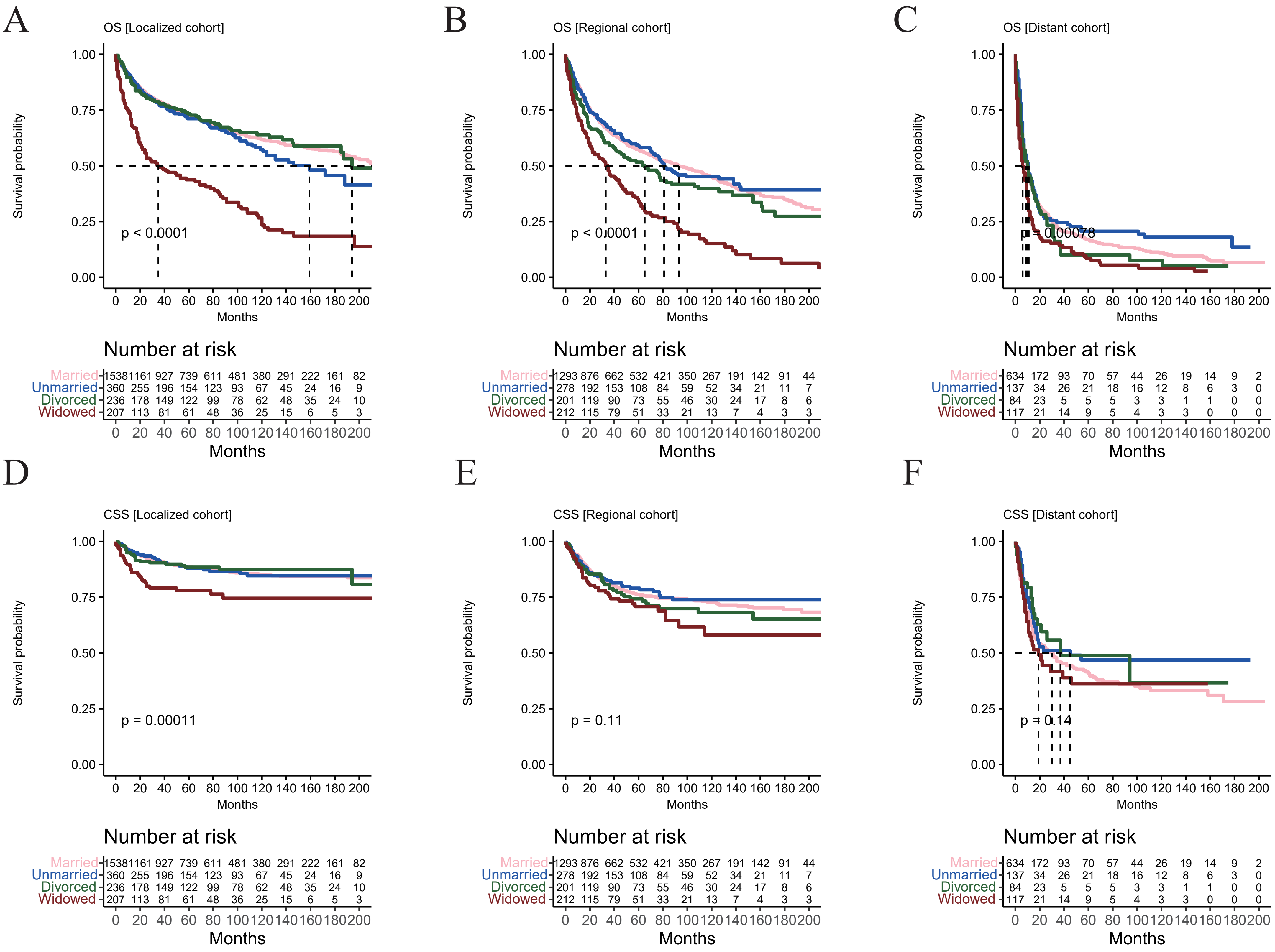

Supplementary Figure 3. Kaplan–Meier curve for analysing the effects of marital status on survival. (A) Overall survival (OS) in localized cohorts; (B) OS in the regional cohort; (C) OS in the distant cohort; (D) Cancer-special survival (CSS) in localized cohorts (E) CSS in the regional cohort; (F) CSS in the distant cohort.
